# 5-Aminosalicylic acid alters the gut microbiota and altered microbiota transmitted vertically to offspring have protective effects against colitis

**DOI:** 10.1038/s41598-023-39491-x

**Published:** 2023-07-28

**Authors:** Haruka Wada, Jun Miyoshi, Satoshi Kuronuma, Yuu Nishinarita, Noriaki Oguri, Noritaka Hibi, Osamu Takeuchi, Yoshihiro Akimoto, Sonny T. M. Lee, Minoru Matsuura, Taku Kobayashi, Toshifumi Hibi, Tadakazu Hisamatsu

**Affiliations:** 1 Department of Gastroenterology and Hepatology, Kyorin University School of Medicine, 6-20-2 Shinkawa, Mitaka, Tokyo 181-8611 Japan; 2 Department of Research, BioMedical Laboratory, Kitasato University Kitasato Institute Hospital, 5-9-1 Shirokane, Minato-ku, Tokyo, 108-8642 Japan; 3 Department of Microscopic Anatomy, Kyorin University School of Medicine, 6-20-2 Shinkawa, Mitaka, Tokyo 181-8611 Japan; 4 Division of Biology, Kansas State University, 136 Ackert Hall, 1717 Claflin Rd, Manhattan, KS 66506 USA; 5 Center for Advanced IBD Research and Treatment, Kitasato University Kitasato Institute Hospital, 5-9-1 Shirokane, Minato-ku, Tokyo, 108-8642 Japan

**Keywords:** Gastrointestinal diseases, Microbiome

## Abstract

Although many therapeutic options are available for inflammatory bowel disease (IBD), 5-aminosalicylic acid (5-ASA) is still the key medication, particularly for ulcerative colitis (UC). However, the mechanism of action of 5-ASA remains unclear. The intestinal microbiota plays an important role in the pathophysiology of IBD, and we hypothesized that 5-ASA alters the intestinal microbiota, which promotes the anti-inflammatory effect of 5-ASA. Because intestinal inflammation affects the gut microbiota and 5-ASA can change the severity of inflammation, assessing the impact of inflammation and 5-ASA on the gut microbiota is not feasible in a clinical study of patients with UC. Therefore, we undertook a translational study to demonstrate a causal link between 5-ASA administration and alterations of the intestinal microbiota. Furthermore, by rigorously controlling environmental confounders and excluding the effect of 5-ASA itself with a vertical transmission model, we observed that the gut microbiota altered by 5-ASA affected host mucosal immunity and decreased susceptibility to dextran sulfate sodium-induce colitis. Although the potential intergenerational transmission of epigenetic changes needs to be considered in this study, these findings suggested that alterations in the intestinal microbiota induced by 5-ASA directed the host immune system towards an anti-inflammatory state, which underlies the mechanism of 5-ASA efficacy.

## Introduction

Inflammatory bowel disease (IBD) is a systemic inflammatory disease presenting with chronic and recurrent relapsing inflammation of the intestinal tract. Two major clinical phenotypes of IBD are ulcerative colitis (UC) and Crohn’s disease. Although the pathophysiology of IBD remains unclear, the intestinal microbiota is now thought to play an important role in the pathogenesis of IBD^1^. The gut microbiota is influenced by various environmental factors^2–4^ and it has a close relationship with the host intestinal immunity^5^. Dysbiosis, an imbalance in the types and numbers of microbes in the intestinal microbiota, has been reported in patients with IBD^6–8^. However, observational studies of IBD patients are limited when investigating causal links between dysbiosis and the development of IBD. Because the pathoetiology of IBD remains unclear, the current therapeutic goal for IBD is to induce and maintain the remission of disease activity. Although the number of therapeutic options for IBD has increased markedly, 5-aminosalicylic acid (5-ASA) is still a key medication for the management of IBD. It is inexpensive and its efficacy and tolerability for induction and maintenance therapy have been proven in clinical practice. Furthermore, 5-ASA is thought to act directly on the local intestinal mucosa, where it is rapidly metabolized^9^. A correlation between the concentration of 5-ASA in the intestinal mucosa and its therapeutic efficacy against IBD has been reported^10^ and several drug-delivery systems have been developed to increase 5-ASA concentrations in target lesions. However, the anti-inflammatory mechanisms of 5-ASA have not been elucidated, although various anti-inflammatory mechanisms have been proposed. Previous studies demonstrated that 5-ASA inhibited cellular damage by suppressing the production of reactive oxygen species^11–13^. Other studies demonstrated that 5-ASA reduced the production of inflammatory cytokines by suppressing the activation of nuclear factor kappa-light-chain-enhancer of activated B cells (NF-κB), which is mediated by tumor necrosis factor-alpha (TNF-α) and interleukin-1 (IL-1) produced by immune cells^14–16^. Furthermore, 5-ASA suppressed the activation of NF-κB via peroxisome proliferator-activated receptor gamma activation by phospholipase D activation^17,18^. Nevertheless, the impact of 5-ASA on the intestinal microbiota, which is thought to be involved in the pathogenesis of IBD, is unclear. A previous study reported that 5-ASA treatment for UC altered the intestinal microbiota^19^; however, a limitation of observational studies that comparing the microbiota in patients with active UC before and after 5-ASA treatment fails to distinguish the direct impact of 5-ASA on the gut microbiota related to the influence of improved disease activity because the intestinal microbiota can change according to the inflammatory conditions^20^.

Here, we hypothesized that one of the mechanisms of the anti-inflammatory effect of 5-ASA is changing the nature of the gut microbiota to an anti-inflammatory state. We tested this hypothesis in a translational study. Investigating a new therapeutic mechanism of 5-ASA via the intestinal microbiota might lead to the development of novel microbial interventions against IBD.

## Results

### Oral administration of 5-aminosalicylic acid alters the gut microbiome

**Figure 1:**
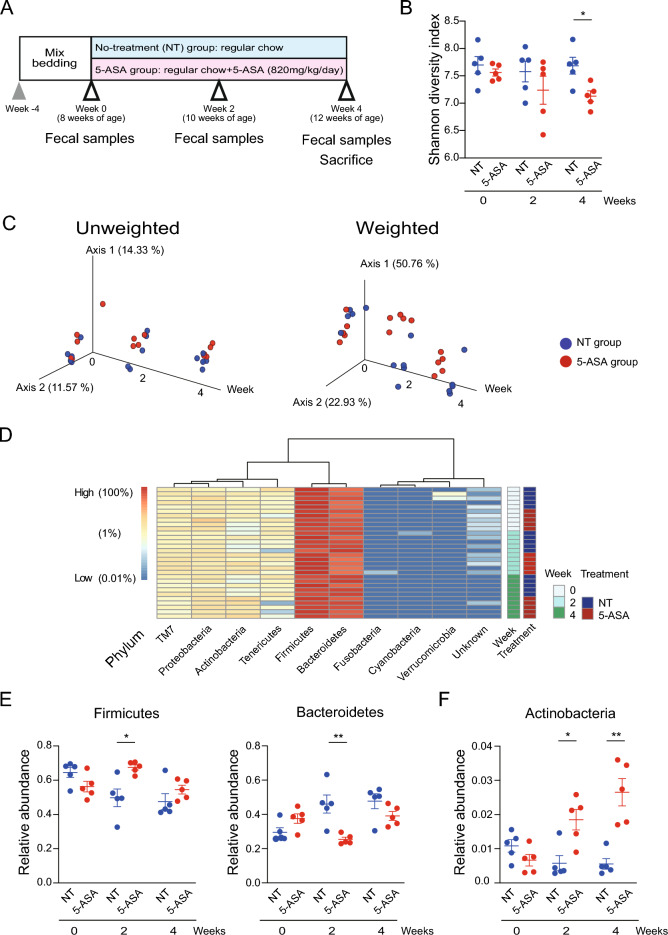
Oral administration of 5-aminosalicylic acid alters the gut bacterial composition. (**A**) The study design of oral 5-aminosalicylic acid (5-ASA) administration. Fecal samples were collected at weeks 0, 2, and 4. Mice were sacrificed to collect tissues at week 4. (**B**) Changes in the Shannon diversity index over time in female animals treated with 5-ASA. (**C**) PCoA plots based on unweighted and weighted UniFrac distances of the intestinal bacterial compositions in female animals. (**D**) Heatmap of relative abundances of bacterial phyla over time in female animals. Each row represents a single DNA sample, and each column represents each phylum. (**E**) Changes in the relative abundances of phyla Firmicutes and Bacteroidetes over time in female animals. (**F**) Changes in the relative abundance of phylum Actinobacteria in female animals. *p < 0.05, **p < 0.01, Mann–Whitney *U-*test. The data are described as the mean ± SEM. Male data are shown in Supplemental Figure S1. Heatmap was generated by Pretty Heatmaps (version 1.0.12) (https://cran.r-project.org/web/packages/pheatmap).

A bedding transfer protocol^21^ was performed between cages to normalize the gut microbiota of mice for 2 weeks before starting 5-ASA administration in a specific-pathogen-free (SPF) environment. The 5-ASA administration protocol is shown in Fig. 1A. The 5-ASA dose for a mouse was equivalent to a clinical dose of 4000 mg/day for a 60 kg person^22,23^. Both 5-ASA and its metabolite-5-acetylamino-2-hydroxybenzoic acid (*N*-acetyl-5-ASA)—were detected in the cecal content of animals in the 5-ASA group by high-performance liquid chromatography (HPLC) (Fig. S1A). Fecal DNA was analyzed by 16S rRNA gene amplicon sequencing. The Shannon diversity index was used to assess alpha diversity and there was no significant difference between the 5-ASA and non-treated (NT) groups at week 0. The Shannon index decreased in the 5-ASA group over time, and it was statistically lower in female animals in the 5-ASA group compared with the NT group at week 4 (*p* = 0.016) (Fig. 1B). A similar tendency was observed in male animals (Fig. S1B). Principal coordinate analysis (PCoA) plots based on unweighted and weighted UniFrac distances showed female samples in the 5-ASA and NT groups changed over time and clustered separately at weeks 2 and 4 (week 0: unweighted *p* = 0.118 and weighted *p* = 0.095, week 2: unweighted *p* = 0.014 and weighted *p* = 0.011, week 4: unweighted *p* = 0.008 and weighted *p* = 0.005) (Fig. 1C). Unweighted UniFrac distances describe the amplicon sequencing variants (ASVs) in the samples, and weighted UniFrac distances describe the proportions of the ASVs. The bacterial composition of male mice in the 5-ASA and NT groups also changed over time and the samples in each group appeared to cluster separately at weeks 2 and 4 (week 0: unweighted *p* = 0.078 and *p* = 0.195, week 2: unweighted *p* = 0.014 and weighted *p* = 0.549, week 4: unweighted *p* = 0.024 and weighted *p* = 0.122) (Fig. S1C).

**Figure 2:**
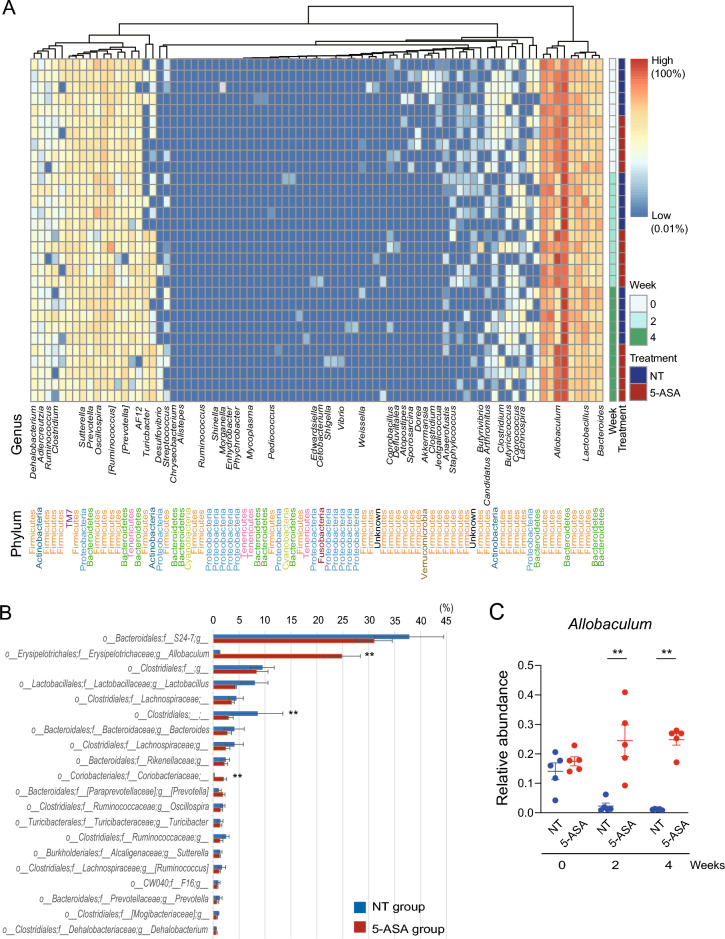
Alterations of bacterial genera by 5-aminosalicylic acid administration. (**A**) Heatmap of the relative abundances of bacterial genera over time in female animals. Each row represents a single DNA sample, and each column represents each genus. A blank in the name of the genus indicates it was not annotated with QIIME 2. (**B**) Top 20 bacterial genera with high relative abundance in female animals treated with 5-aminosalicylic acid (5-ASA). (**C**) Relative abundance of the genus *Allobaculum* over time in female animals. **p < 0.01, Mann–Whitney *U-*test. Corrected *p* was not significant with Mann–Whitney *U*-test and Benjamini–Hochberg procedure. The data are described as the mean ± SEM. Male data are shown in Supplemental Figure S2. Heatmap was generated by Pretty Heatmaps (version 1.0.12) (https://cran.r-project.org/web/packages/pheatmap).

The relative abundances of phyla in female and male animals are presented in Fig. 1D and Fig. S1D, respectively. Firmicutes and Bacteroidetes were the dominant phyla in the cohorts in this study. In female animals, the relative abundance of Firmicutes was significantly higher (*p* = 0.016) and the relative abundance of Bacteroidetes was significantly lower (*p* = 0.008) in the 5-ASA group compared with the NT group at week 2. The higher relative abundance of Firmicutes and lower relative abundance of Bacteroidetes in the 5-ASA group were also observed at week 4 (Fig. 1E). In addition, the relative abundance of Actinobacteria was significantly increased at weeks 2 and 4 in the 5-ASA group compared with the NT group (week 2: *p* = 0.032, week 4: *p* = 0.008) (Fig. 1F). A similar change in Firmicutes and Bacteroides after 5-ASA treatment was observed in male animals at weeks 2 and 4 (Fig. S1E) although this was not as apparent as in female animals. Furthermore, Actinobacteria increased over time in the 5-ASA group and there was a significant difference in the relative abundance between the 5-ASA and NT groups (*p* = 0.048) (Fig. S1F). The relative abundances of genera in female and male animals are presented in Fig. 2A and Fig. S2A, respectively. The relative abundances of ASV in each sex are shown in Fig. S3. The 20 genera with the highest relative abundance in female and male animals in the 5-ASA group at week 4 are shown in Fig. 2B and Fig. S2B, respectively. In female animals, the relative abundance of *Allobaculum* was significantly higher in the 5-ASA group (*p* = 0.008) compared with the NT group (Fig. 2C) and this difference in the relative abundance between the groups was the largest among the genera (Fig. 2B). *Allobaculum* were also increased in male animals in the 5-ASA group (Fig. S2C) and this difference in the relative abundance between the groups was the largest among the genera (Fig. S2B).

### Impact of oral 5-aminosalicylic acid administration on intestinal morphology and the mucosal immune system

**Figure 3:**
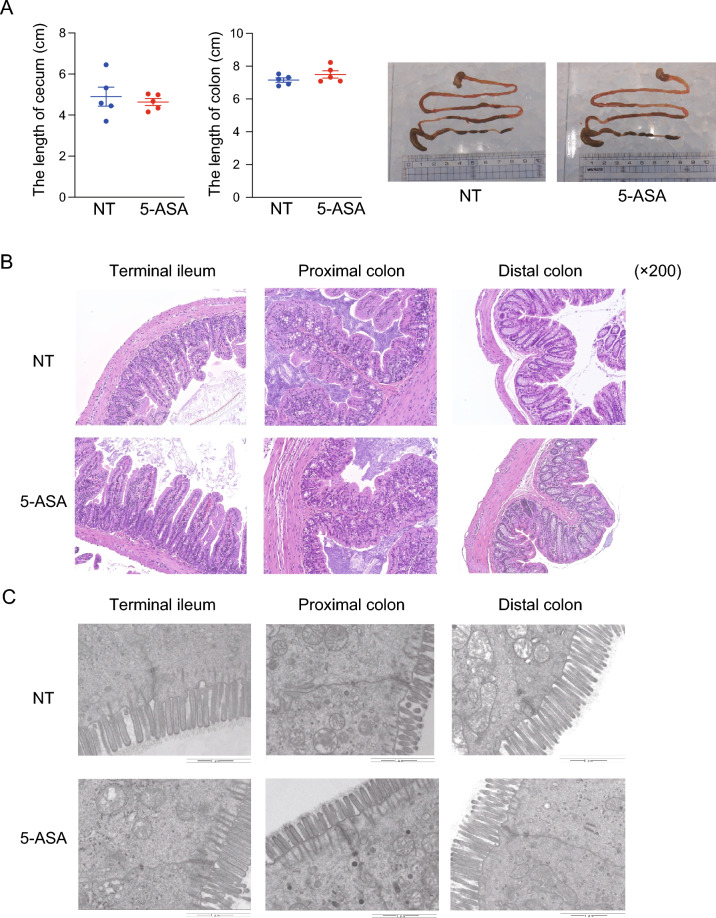
Oral 5-aminosalicylic acid administration does not affect the intestinal morphology. (**A**) The length of the cecum and colon in female animals in the 5-aminosalicylic acid (5-ASA) group and the non-treated (NT) group. Representative images are presented. (**B**) Representative images of female intestinal specimens stained with hematoxylin and eosin (H&E) under an optical microscope (×200). (**C**) Representative images of female intestinal specimens obtained by transmission electron microscopy. Scale bars 1 µm (see Supplemental Figure S4D). The data are described as the mean ± SEM. Male data are shown in Supplemental Figure S4.

**Figure 4:**
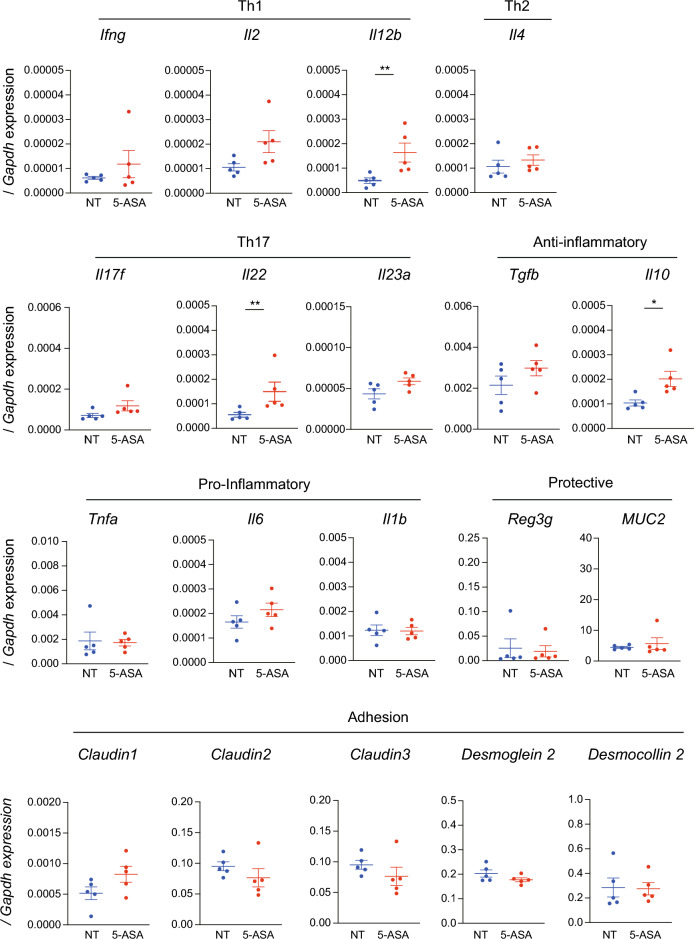
The oral administration of 5-aminosalicylic acid influences mRNA expression in the colonic mucosa. mRNA expressions of cytokines and pro- and anti-inflammatory molecules involved in colonic inflammation in the colonic mucosa were examined in the 5-aminosalicylic acid (5-ASA) group and non-treated (NT) group by real-time qPCR. mRNA expressions are expressed as ΔΔCT relative to the housekeeper gene *Gapdh*. Female data are presented as the mean ± SEM. *p < 0.05, **p < 0.01, Mann–Whitney *U-*test. Male data are shown in Supplemental Figure S5.

The macroscopic morphology, including the length of the cecum and colon in either sex, was not different between the 5-ASA and NT groups (Fig. 3A, Fig. S4A). In the microscopic assessment of hematoxylin and eosin (H&E)-stained intestinal specimens (terminal ileum, proximal colon, and distal colon), no apparent difference in either sex was observed between the 5-ASA and NT groups (Fig. 3B, Fig. S4B). An analysis of the ultrastructure of the intestinal mucosal epithelium assessed by transmission electron microscopy (TEM) (Fig. 3C, Fig. S4C) indicated no significant differences in the length of microvilli, length of tight junctions, and length and width of the adherens junctions in either sex between the 5-ASA and NT groups (Fig. S4D). These results indicated that 5-ASA treatment did not cause morphological changes in the intestinal mucosal epithelium. Next, the mRNA expressions of inflammatory/anti-inflammatory cytokines, anti-microbial/protective factors, and intercellular adhesion molecules in the colonic mucosa were examined to determine the influence of oral 5-ASA administration on the host intestinal immune system, including the mucosal barrier function. The expression of *Il10* in both sexes was significantly higher in the 5-ASA group compared with the NT group (female: *p* = 0.016, male: *p* = 0.032). *Il22* expression was also increased significantly in both sexes (female: *p* = 0.008, male: *p* = 0.032). In female animals, the expression of *Il12b* was higher in the 5-ASA group than in the NT group (*p* = 0.008) (Fig. 4, Fig. S5). There were no significant differences in the mRNA expressions of anti-microbial/protective factors such as Reg3γ and Muc2 between the 5-ASA and NT groups (Fig. 4, Fig. S5). The expressions of *Claudin 1*, *Claudin 2*, and *Claudin 3* (components of the tight junction) and *Desmoglein* 2 and *Desmocollin* 2 (components of the adherens junction) were not different between the 5-ASA and NT groups (Fig. 4, Fig. S5).

### A vertical transmission model of gut microbiota altered by 5-aminosalicylic acid and the impact of the altered microbiota on the host mucosal immune system

**Figure 5:**
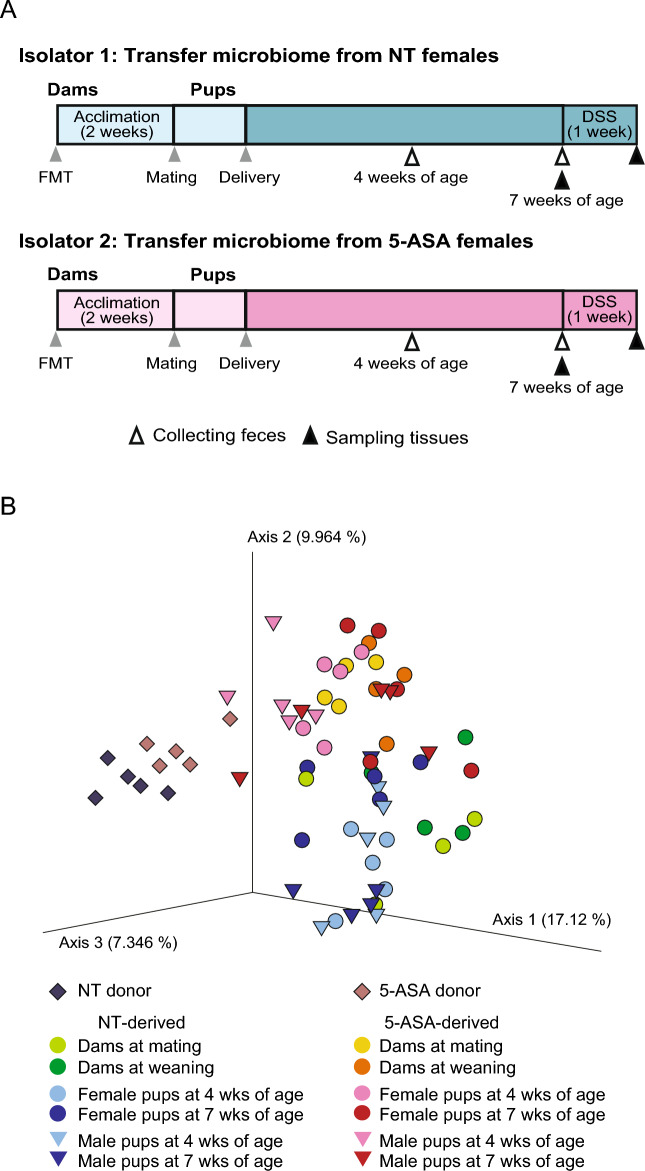
Vertical transmission of the gut microbiota in non-treated mice or mice treated with 5-aminosalicylic acid. (**A**) The study design took advantage of the intergenerational vertical transmission of intestinal microbiota to obtain pups with gut microbiota treated with 5-aminosalicylic acid (5-ASA) whilst excluding the possible effect of 5-ASA residues. Five pups of each sex were sacrificed at 7 weeks of age for further analyses and the others were used for dextran sodium sulfate-induced colitis experiments. (**B**) PCoA plot based on unweighted UniFrac distances of bacterial compositions of animals in Isolator 1 (the non-treated [NT]-derived group) and Isolator 2 (the 5-ASA-derived group), and the donors of fecal microbiota transfer.

Next, we examined the influence of the gut microbiota altered by 5-ASA on the host mucosal immune system, excluding the possible impact of the presence of 5-ASA residues in the intestinal tract. First, fecal microbiota transfer (FMT) was performed in 12-week-old female germ-free (GF) mice using fecal samples collected from 12-week-old female SPF mice in separate isolators with/without 4-week oral 5-ASA administration (Fig. 5A). Then, the five FMT recipient female mice in each isolator (NT-derived group and 5-ASA-derived group) were mated with GF male mice 2 weeks after FMT and these breeding pairs were housed in separate isolators. Consequently, 23 pups (13 female and 10 male) from dams of the NT-derived group and 30 pups (14 female and 16 male) from dams of the 5-ASA-derived group were obtained. The pups were weaned at 4 weeks of age and continued to be housed in each isolator. In the PCoA plot of bacterial compositions based on unweighted UniFrac distances, dams in the NT-derived and 5-ASA-derived groups appeared to cluster separately at mating and weaning, respectively (mating: *p* = 0.024, weaning: *p* = 0.027) (Fig. 5B). The dams and pups in each group clustered closely at weaning in the PCoA plot, suggesting the vertical transmission of the gut microbiome from dams to pups in each isolator. There was a significant difference between the 5-ASA-derived group (dams and pups) versus the NT-derived group (dams and pups) in unweighted UniFrac distances (*p* = 0.001) (Fig. 5B). In the PCoA plot based on unweighted UniFrac distances, pups of both sexes in the 5-ASA-derived and NT-derived groups clustered separately at 4 and 7 weeks of age (4 weeks of age: female *p* = 0.008, male *p* = 0.004; 7 weeks of age: female p = 0.004, male p = 0.016) (Fig. 5B). Whereas the FMT donors and other samples appeared separate, among 30 genera with an average relative abundance > 0.1% in the NT donor samples, 24 genera (80.0%) were detected in recipient dams at mating and, among 31 genera with an average relative abundance > 0.1% in the 5-ASA donor samples, 28 genera (90.3%) were detected in recipient dams at mating. Among the 30 genera in the NT donor samples, 22 genera (73.3%) and 24 genera (80%) were detected in female and male pups at 7 weeks of age, respectively, in the NT-derived group. Among the 31 genera in the 5-ASA donor, 24 genera (77.4%) and 28 genera (90.3%) were detected in female and male pups at 7 weeks of age, respectively, in the 5-ASA-derived group.

**Figure 6:**
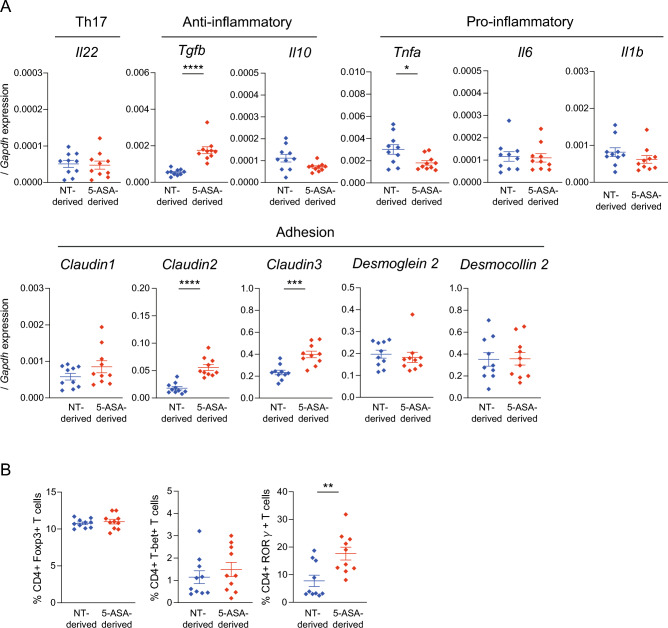
Vertically transmitted 5-aminosalicylic acid-induced alterations of the intestinal microbiota influence the host immune profile. (**A**) mRNA expressions in the colonic mucosa of the 5-aminosalicylic acid (5-ASA)-derived group and non-treated (NT)-derived group were examined by real-time qPCR (n = 10). mRNA expressions are expressed as ΔΔCT relative to the housekeeper gene *Gapdh*. (**B**) Flow-cytometric analyses of live CD45+ TCRβ^+^CD4^+^ T cells expressing Foxp3^+^, T-bet^+^, or RORγt^+^ in the mesenteric lymph nodes of the 5-ASA-derived and NT groups (n = 10). The data are the mean ± SEM. *p < 0.05, **p < 0.01, ***p < 0.001, ****p < 0.0001, Mann–Whitney *U*-test. The gating strategy for flow cytometric analyses is shown in Supplemental Figure S8.

At 7 weeks of age, five female and five male pups in each group were sacrificed to assess the impact of the gut microbiota altered by 5-ASA on the intestinal morphology and mucosal immune system. The macroscopic morphology in each sex, including the length of the cecum and colon, was not different between the 5-ASA-derived and NT-derived groups at 7 weeks of age (Fig. S6A). There was no apparent difference in the microscopic assessment of H&E-stained intestinal specimens (terminal ileum, proximal colon, and distal colon) from either sex between the 5-ASA-derived and NT-derived groups (Fig. S6B). Assessment of the ultrastructure of the intestinal mucosal epithelium by TEM (Fig. S6C) indicated no significant differences in the length of microvilli, length of tight junctions, and length and width of adherens junctions in either sex between the 5-ASA-derived and NT-derived groups (Fig. S7). These results indicated that the vertically transmitted gut microbiota altered by 5-ASA did not cause morphological changes in the intestinal mucosal epithelium. The mRNA expressions of inflammatory/anti-inflammatory cytokines, anti-microbial/protective factors, and intercellular adhesion molecules in the colonic mucosa were examined to evaluate the influence of the gut microbiota altered by 5-ASA on the host intestinal immune system, including the mucosal barrier function (Fig. 6A). The expression of *Tgfb* was significantly higher in the 5-ASA-derived group than in the NT-derived group (*p* < 0.0001). The expression of *Tnfa* was significantly lower in the 5-ASA-derived group than in the NT-derived group (*p* = 0.043). The expressions of *Claudin 2* and *Claudin 3* were increased significantly in the 5-ASA-derived group compared with the NT-derived group (*Claudin 2*: *p* < 0.0001, *Claudin 3*: *p* < 0.001). There were no significant differences in the mRNA expressions of anti-microbial/protective factors between the 5-ASA-derived and NT-derived groups (Fig. 6A). CD4^+^ T cell populations in the mesenteric lymph nodes (MLNs) were assessed by flow cytometry. The gating strategy is shown in Fig. S8. CD4^+^RORγt^+^ cells were increased in the 5-ASA-derived group (17.6 ± 2.2%) compared with the NT-derived group (7.8 ± 1.9%) (*p* = 0.009) (Fig. 6B).

### Protective effect of the vertically transmitted gut microbiota altered by 5-aminosalicylic acid on murine experimental colitis

**Figure 7:**
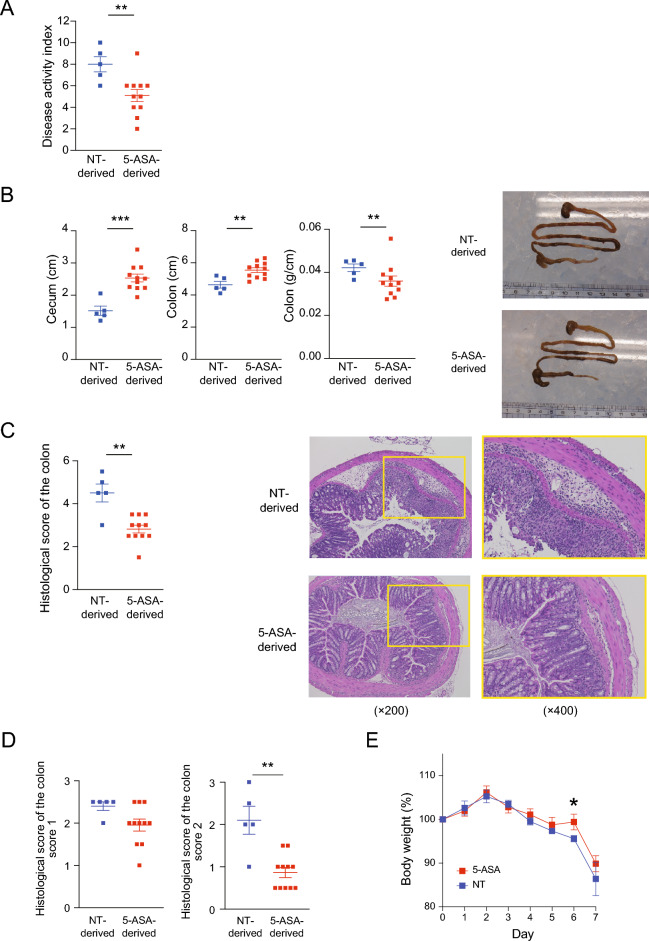
Vertical transmission of intestinal microbiota altered by 5-aminosalicylic acid leads to a protective effect protect against dextran sulfate sodium-induced colitis. The severity of colitis was assessed on day 7 after dextran sulfate sodium (DSS)-induced colitis was initiated in the 5-aminosalicylic acid (5-ASA)-derived group and non-treated (NT)-derived group. Male data are shown (n = 5 in the NT-derived group and n = 11 in the 5-ASA-derived group) (**A**) disease activity index. (**B**) The length of the cecum, length of the colon, and the colon weight per length with representative macroscopic images. (**C**) Histological score of the colon with representative microscopic images (×200 and ×400). (**D**) Histological scores of score 1 (inflammatory cell infiltrate) and score 2 (intestinal architecture). (**E**) Body weight changes during DSS treatment. The data are the mean ± SEM. **p < 0.01, ***p < 0.001, Mann–Whitney *U-*test. Female data are shown in Supplemental Figure S9.

Pups at 7 weeks of age in each isolator were used for dextran sulfate sodium (DSS)-induced colitis experiments. A 2.5% DSS solution was orally administered for 7 days and mice were sacrificed on day 7. The disease activity index (DAI)^24^ at day 7 was significantly lower in the 5-ASA-derived group (5.1 ± 0.5) compared with the NT-derived group (8.0 ± 0.6) in male animals (*p* = 0.009) (Fig. 7A). A similar tendency was observed in female animals (Fig. S9A). The length of the cecum of male animals in the NT-derived group (1.52 ± 0.13 cm) was significantly shorter compared with the 5-ASA-derived group (2.53 ± 0.12 cm) (*p* = 0.001) (Fig. 7B). The length of the colon of male animals in the NT-derived group (4.64 ± 0.42 cm) was also significantly shorter compared with the 5-ASA-derived group (5.54 ± 0.14 cm) (*p* = 0.009) (Fig. 7B). Similar tendencies were observed in female animals (Fig. S9B). The colon weight per length was heavier in male animals in the NT-derived group (0.042 ± 0.002 g/cm) compared with those in the 5-ASA-derived group (0.036 ± 0.002 g/cm) (*p* = 0.004), indicating the NT-derived group had more severe colitis than the 5-ASA-derived group (Fig. 7B). This difference was not apparent in female animals (Fig. S9B). Representative macroscopic images of the gastrointestinal tract are shown in Fig. 7B and Fig. S9B. The histological disease activity scores of colon specimens^25^ were significantly lower in male animals (*p* = 0.007) in the 5-ASA group (2.7 ± 0.2) than in those in the NT group (3.3 ± 0.2) (Fig. 7C). A similar tendency was observed in female animals (Fig. S9C). Representative microscopic images of colonic specimens are shown in Fig. 7C and Fig. S9C. Regarding histological scoring, score 1 (inflammatory cell infiltrate) and score 2 (intestinal architecture) were lower for male animals in the 5-ASA group than for those in the NT group and a statistical difference was observed for score 2 (*p* = 0.002) (Fig. 7D). A similar tendency was observed in female animals (Fig. S9D). Furthermore, the NT-derived group tended to lose more weight compared with the 5-ASA-derived group during the DSS treatment (Fig. 7E, Fig. S9E). Taken together, the clinical findings and macroscopic and microscopic findings of the colon showed that intestinal inflammation induced by DSS was milder in the 5-ASA group compared with the NT group, particularly in male animals. Furthermore, our findings were in accord with a previous study^26^ reporting that female mice were less susceptible to DSS-induced colitis compared with male mice.

## Discussion

We observed that orally administered 5-ASA reached the colon and altered the intestinal bacterial compositions of mice without intestinal inflammation. This translational study demonstrated the potential of 5-ASA to change the gut microbiome. In a previously reported study of patients with UC^19^, a causal relationship between 5-ASA administration and alteration of the intestinal microbiota composition was unclear because of the confounding effects of inflammation. Because performing interventional studies where 5-ASA is orally administered to healthy individuals is difficult, our study design took advantage of animal experiments. The alpha diversity of intestinal bacteria in mice decreased after 5-ASA administration in this study. This finding suggests that bacteria in the intestinal environment were selected by their survival and growth attributes in the presence of 5-ASA. At the phylum level, we observed an increase in Firmicutes and a decrease in Bacteroidetes. These tendencies were consistent with a previous observational study in patients with UC^19^, although the alterations in that report might have been related to the impact of 5-ASA administration on the disease activity. In our study, the genus *Allobaculum* of the phylum Firmicutes was increased in the 5-ASA group compared with the NT group. Because *Allobaculum* produces short-chain fatty acids (SCFAs)^27^, which are crucial for intestinal homeostasis including maintaining intestinal epithelial cells and regulating immune functions^28^, this change may have contributed to the suppression of inflammation via the increase of SCFAs. Further metabolomic analyses will illuminate the physiological significance of our findings. The phylum Actinobacteria, which was increased in the 5-ASA group, includes *Bifidobacterium* and *Propionibacterium,* which are considered potential probiotics^29,30^. Previous studies reported that Actinobacteria are useful for maintaining remission in UC^31,32^. Another study demonstrated that Actinobacteria were increased in patients with IBD^33^, although the effects of inflammation and therapeutic interventions need to be considered because the study analyzed real-world patients with active disease. In addition to the challenge of distinguishing between the impacts of inflammation and drugs on the microbiota, another explanation for these apparently contradictory findings is that a bacterial phylum includes a variety of genera, species, and strains, and thus, a phylum cannot be simply classified as inflammatory or anti-inflammatory. The gut microbiota and host immune systems interact closely^5^. We observed that the mRNA expressions of *Il10*, *Il22*, and *Il12b* were increased in the colonic mucosa after 5-ASA administration compared with the NT group. IL-10 is a major anti-inflammatory cytokine that suppresses the production of inflammatory cytokines and the functions of antigen-presenting cells^34–36^. IL-10 mutations cause very-early-onset IBD^37^ and IL-10 deficient mice are widely used as an IBD animal model^38^. Thus, 5-ASA-induced *Il10* expression in the colonic mucosa might have anti-inflammatory effects. IL-22 plays a role in mucosal defense by promoting epithelial regeneration and increasing the expressions of antimicrobial proteins^39–41^. The increase in *Il22* expression suggests its contribution to maintaining mucosal defense. The *Il12b* gene encodes a subunit (p40) of IL-12 and IL-23, which are involved in the differentiation, induction, and maintenance of Th1 and Th17 cells^42–45^. However, there were no significant changes in *Ifng* and *Il17f*, which are representative cytokines produced by Th1 and Th17 cells, or other inflammatory cytokines such as *Tnfa*, *Il6*, and *Il1b* in our study. These findings suggest that the observed alterations in *Il12b* expression did not cause pathological effects. Overall, the experiments using healthy mice demonstrated the potential of 5-ASA to alter the intestinal bacterial composition and mucosal immune system.

The aim of this study was to investigate a causal link between alterations in the intestinal microbiota mediated by 5-ASA and its protective effect against DSS-induced colitis, a widely used murine IBD model. For this purpose, we carefully excluded the possibility that 5-ASA and/or its metabolites reaching the colon exerted a direct anti-inflammatory effect in the intestine. In this study, we took advantage of the intergenerational vertical transmission of intestinal microbiota^46^. That is, the intestinal microbiota altered by 5-ASA administration or the intestinal microbiota of the NT group was transferred to GF dams, which were then mated with germ-free sires, and their pups were used for further analyses in the 5-ASA-derived or NT-derived groups, respectively. We prepared a vinyl isolator for each group and performed all experiments in each isolator and the vertical transmission of the bacterial composition between dams and pups in each isolator was confirmed. This study design with strict microbiome control and the exclusion of 5-ASA and its metabolites underscores the scientific significance of this study. Although the exposure to 5-ASA and its metabolites in dams was considered minimal only in the FMT procedure, we cannot rule out the possibility that some epigenetic changes were caused in the dams by 5-ASA-induced alterations of the gut microbiota, and that these changes were transmitted to the pups affecting their immunological profiles and clinical outcomes. The potential intergenerational transmission of epigenetic changes is an unavoidable limitation of microbiome studies using the vertical transmission model. Because epigenetic changes can be confounding factors when assessing the effects of gut microbiota, we should interpret the data in the present study with an understanding of this limitation.

In the analysis of mRNA expression in the colonic mucosa, we observed a decrease in *Tnfa* and an increase in *Tgfb*, suggesting the anti-inflammatory effect of the 5-ASA-induced alterations of the gut microbiota. A previous study reported that 5-ASA decreased the production of *Tnfa* by suppressing NF-κB pathway activation^16^. Furthermore, 5-ASA administration was reported to induce regulatory T cells (Treg) and, transforming growth factor (TGF)-β expression in the colonic mucosa^47^. Our results suggest that alterations of the intestinal microbiota by 5-ASA might be involved in these mechanisms in the colon. The mRNA expressions of *Claudin2* and *Claudin3*, constituent proteins of tight junctions contributing to mucosal defense, were increased in this study. Claudin-2 is a cation and water channel and is involved in the regulation of the water content and Na concentration in the lumen^48^. Intestinal inflammation was reported to be more severe in Claudin-2 deficient mice compared with wild-type mice^49,50^. Other studies indicated that decreased *Claudin2* expression correlated with increased levels of inflammatory cytokines, such as IL-6 and IL-1β, leading to more severe intestinal inflammation^49,51^. Claudin-2 was also reported to protect against inflammation in association with increased TGF-β expression^52^, suggesting Claudin-2 plays an important role in intestinal homeostasis. Claudin-3 is thought to enhance adhesion^53,54^, and its increased expression might improve mechanical defense. These findings suggest the protective mechanism of intestinal microbiota altered by 5-ASA might be associated with its effects on epithelial defense. Other claudin proteins thought to be related to IBD, such as Claudin-4, Claudin-7, and Claudin-12, might be future study targets. The present study demonstrated that a subpopulation of CD4^+^RORγ^+^ T cells was increased in the mesenteric lymph nodes of pups from dams with a gut microbiota altered by 5-ASA. Although we assessed Foxp3, T-bet, and RORγt, typical nuclear factors characteristic of Treg, Th1, and Th17 cells, respectively, CD4^+^ T cells have plasticity, and populations of these cells express multiple nuclear factors. Therefore, further immunological studies are needed to understand this finding. Furthermore, the inflammatory state in the MLN might not always reflect the local immunological properties in the colonic mucosa. A previous study showed that 5-ASA administration induced active TGF-β in the colonic mucosa although a subpopulation considered as Treg in the MLNs did not increase^47^. The present study demonstrated the offspring that underwent 5-ASA-induced alterations of intestinal microbiota from their dams were less susceptible to DSS-induced colitis than the controls. This clinical finding seems consistent with the results of our immunological analyses. Because we used a vertical transmission model, we cannot strictly distinguish the effect of alterations of the gut microbiota by 5-ASA and potential epigenetic changes. Nevertheless, a major advantage of this study design was that we obtained insights into the clinical impact of the 5-ASA-induced alterations of the gut microbiota, while excluding the direct effect of 5-ASA. Male animals have been reported to be more susceptible to DSS-induced colitis and develop more severe colitis compared with female animals^26^. We speculate this sex difference might explain why the beneficial effect of the intestinal microbiota altered by 5-ASA was more apparent in male mice compared with female mice. Indeed, colitis was not severe in female animals, even in the control group, and there were no statistical differences in severity between female animals in the treatment and control groups.

The present study had several limitations. First, it is challenging to reproduce the bacterial compositions of donor animals (including the balance between aerobic and anaerobic bacteria) in recipient animals. Performing all FMT procedures in an anaerobic environment is not possible. Furthermore, although the gavage procedure is widely accepted for FMT, we cannot exclude the influence of digestive fluids in the gastrointestinal tract. In addition, although we strictly controlled the intestinal microbiota using GF animals and isolators, we could not exclude the possibility that the microbiota in each isolator changed slightly over time during the experiment. Another limitation is differences in the intestinal microbiota between host species. Although this translational research using murine models investigated a causal link beyond the association observed in the intestinal microbiota study, the gut microbiota can vary depending on the host species^55^ and it remains unclear whether the bacteria altered by 5-ASA in mice (e.g., *Allobaculum*) are also changed by 5-ASA in humans, particularly in patients with IBD. Further clinical studies are necessary to identify bacteria affected by 5-ASA and to investigate whether they might be a target for microbial interventions in IBD. A recent study with human subjects demonstrated that gut microbial metabolism impacted the inactivation of 5-ASA leading to an increased risk of treatment failure with 5-ASA^56^. Their findings and our results underscore the importance of understanding the close, crucial interactions of 5-ASA and the gut microbiota in human IBD. In addition, future studies should investigate the physiological significance of changes in the host immune system by 5-ASA-induced altered gut microbiota. Nevertheless, we think this study provides proof of the concept that 5-ASA can alter the gut microbiota, which affects host immunity leading to a protective effect against colitis.

In conclusion, the present study demonstrated that oral 5-ASA administration to mice altered the bacterial composition of their intestinal microbiota, which decreased the susceptibility of mice to colitis in offspring in the vertical transmission model. These findings suggest that altering the intestinal microbiota might be part of the mechanism involved in the anti-inflammatory effects of 5-ASA.

## Methods

### Animals

SPF C57BL/6 wild-type mice were purchased from CLEA Japan (Tokyo, Japan). Mice were maintained and treated under SPF conditions. GF mice were purchased from Sankyo Labo Service Corporation (Tokyo, Japan) and housed and treated in a gnotobiotic isolator. SPF and GF mice were fed gamma sterilized or autoclaved CLEA Rodent Diet CE-2 (CLEA Japan), respectively. All mice were supplied with drinking water ad libitum. The bedding transfer protocol^21^ was performed twice a week to normalize the gut microbiota before each experiment was initiated. All mice were euthanized at the end of the study by carbon dioxide inhalation and cervical dislocation. Experiments were reviewed and approved by the Institutional Animal Care and Use Committee for Kitasato University (Approval numbers 19-041, 20-009, 20-009-2, and 21010). All animal experiments were conducted according to the Japanese guidelines for experimental animal welfare and study protocols. Authors have complied with the ARRIVE guidelines for reporting.

### 5-Aminosalicylic acid treatment

5-ASA was obtained from Sigma-Aldrich (St. Louis, MO, USA) and mixed with powdered CLEA Rodent Diet CE-2 (CLEA Japan). The dosage of 5-ASA was calculated based on the normalization method using the body surface area. The determined dose for a mouse was 820 mg/kg/day, which was equivalent to a dose of 4000 mg for a 60 kg person^22,23^. Considering the daily food intake of an adult C57BL/6 mouse^57^, we used 0.4% 5-ASA as we did in the previous study^23^. Mice were treated with 5-ASA for 4 weeks from 8 to 12 weeks of age. The control group received powdered CLEA Rodent Diet CE-2 (CLEA Japan).

### High-performance liquid chromatography

HPLC was performed using cecal contents to determine the presence of 5-ASA and its primary metabolite, *N*-acetyl-5-ASA as previously described^58^. The mobile phase consisted of 0.1% formic acid and acetonitrile containing 0.1% formic acid (50:50). Then, 20 mg of cecal contents were weighed and dissolved in 400 μL of the mobile phase. The samples were centrifuged at 17,800×*g* for 5 min at room temperature and filtered through a Cosmonice filter W (pore size 0.45 μm) (Nacalai Tesque, Kyoto, Japan). Chromaster 5430 (Hitachi High-Tech Corporation, Tokyo, Japan), Lichrospher 100 RP-18 endcapped (5 μm) LichroCART 150–4.6 (Merck KGaA, Darmstadt, Germany), and Manu-CART NT cartridge holders (Merck KGaA) were used for HPLC. As controls, 1 mg/ml solutions of 5-ASA (Sigma-Aldrich) and *N*-acetyl-5-ASA (Sigma-Aldrich) were used. The detection wavelength was set at 298 nm for 5-ASA and 313 nm for *N*-acetyl-5-ASA^59^.

### Histological analysis

Colon tissue was fixed in a 4% formalin solution and embedded in paraffin. Tissue sections were stained with hematoxylin and eosin (H&E) and examined by light microscopy. A previously reported scoring system for the histopathological evaluation of colitis^25^ was used in this study (Supplemental Table 1).

### Transmission electron microscopy

Collected colon tissue samples were washed with phosphate-buffered saline (PBS) and pre-fixed in 2.5% glutaraldehyde (Nissin EM, Tokyo, Japan) with PBS. Post-fixation was performed with 1% osmium tetroxide (Nissin EM) in 0.1 M phosphate buffer (pH 7.2). The samples were embedded in EPON resin (Nissin EM) and cut as ultrathin sections (80 nm). The ultrathin sections were electronically stained with 2% uranium acetate solution followed by 1% lead citrate solution. The sections were observed by TEM (JEM-1011, JEOL, Tokyo, Japan). Ultrastructural findings, including the microvilli length and intercellular adhesion, were assessed on the captured images.

### Fecal microbiota transplantation

Fecal samples were collected from female mice treated with 5-ASA (5-ASA group) and non-treated female mice (NT group) at 12 weeks of age. A fecal solution was prepared by dissolving 100 mg of feces in 1 mL of PBS for FMT. In isolators, the fecal solution (200 µL) was gavaged into female GF mice at 12 weeks of age. The recipients in the 5-ASA group and NT group were housed in separate isolators during the observation period.

### Dextran sulfate sodium-induced colitis

Mice were administered 2.5% DSS (molecular weight 36,000–50,000 Da) (MP Biomedicals, Santa Ana, CA, USA) dissolved in distilled drinking water ad libitum for 7 days. After the treatment, mice were euthanized and collected tissues were analyzed.

### Disease activity index

The DAI for the murine DSS-induced model was assessed using a scoring system previously described^24^ on day 7 before sacrifice. The scoring system includes weight loss, stool consistency, and bleeding as evaluation items (Supplemental Table 2).

### DNA extraction and 16S rRNA gene amplicon sequencing analysis

Fecal samples were collected and frozen at − 80 °C. DNA was extracted from the fecal samples as previously described^46^. Following the amplification of the 16S rRNA gene V4 region, amplicon sequencing analysis was performed by Miseq (Illumina, San Diego, CA, USA). The sequences were processed using the Quantitative Insights into Microbiome Ecology (QIIME) 2 pipeline^60^. Sequences were denoised and filtered using DADA2^61^. The sampling depth was set as 5000. Amplicon sequence variants (ASVs) were assigned to taxonomy using the Greengenes database 13_8 (https://docs.qiime2.org/2021.2/data-resources/). The diversity of the bacterial community was assessed with QIIME 2. Alpha diversity was evaluated using the Shannon diversity index. Beta diversity was assessed with UniFrac distances and visualized using principal coordinates analysis (PCoA).

### Reverse-transcription quantitative PCR

Total RNA extraction from the colic mucosa was performed using TRIzol Reagent (Thermo Fisher Scientific, Waltham, MA, USA) following the manufacturer’s protocol. First-strand cDNA was synthesized using a High-Capacity cDNA Reverse Transcription Kit (Thermo Fisher Scientific) following the manufacturer’s protocol. Quantitative PCR was performed using a PowerUp SYBR Green Master Mix (Thermo Fisher Scientific) with QuantStudio 5 (Thermo Fisher Scientific) following the manufacturer’s protocol. The primers were designed based on PrimerBank (https://pga.mgh.harvard.edu/primerbank/). Primers used for qPCR are listed in Supplemental Table 3.

### Flow cytometry

T-cell populations were analyzed by flow cytometry using procedures previously described^46^. Briefly, MLNs were harvested, homogenized, and resuspended in PBS containing 2% fetal bovine serum. For all samples, Fc receptor blocking was performed with Purified Rat Anti-Mouse CD16/CD32 (BD Biosciences, San Jose, CA, USA). Cells were stained using a LIVE/DEAD Fixable Aqua Dead Cell Stain Kit (Thermo Fisher Scientific) to assess viability. Anti-mouse CD45 (BioLegend, San Diego, CA, USA), anti-mouse TCRβ (BioLegend), and anti-mouse CD4 (BioLegend) antibodies were used for surface staining. A FOXP3/Transcription Factor Staining Buffer Set (Thermo Fisher Scientific) was used to fix and permeabilize cells. Anti-mouse/rat Foxp3 (Thermo Fisher Scientific), anti-mouse/human T-bet (Thermo Fisher Scientific), and anti-Mouse RORγt (Thermo Fisher Scientific) antibodies were used for intranuclear staining. Mouse IgG1 Kappa Isotype Control (Thermo Fisher Scientific), Rat IgG1 Isotype Control (Thermo Fisher Scientific), and Rat IgG2a Kappa Isotype Control (Thermo Fisher Scientific) were used as isotype controls. Samples were analyzed with CytoFLEX (Beckman Coulter, Brea, CA, USA) and FlowJo version 10.8 (FLOWJO, Ashland, OR, USA).

### Statistical analysis

The Mann–Whitney *U-*test was performed using GraphPad Prism version 8.4.3 to compare the body weights, colon weights, colon lengths, colon weight/length ratios, Shannon diversity indexes, T cell populations, mRNA expressions, or histological scores between two groups. Mann–Whitney *U-*test and Benjamini–Hochberg procedure were performed with R (version 4.1.3) (https://www.R-project.org/) to compare the relative abundances of genera between the groups. PERMANOVA analyses were performed with QIIME 2^60^. The criterion of statistical significance was set as a *p *value < 0.05 or *q *value < 0.05.

## Supplementary Information

Supplementary Information.

## Data Availability

The accession number of the microbial dataset and the mouse sample information reported in this paper is DRA: DRA015730 (https://www.ddbj.nig.ac.jp/dra/). The datasets generated during and/or analyzed during the current study are available from the corresponding author on reasonable request.

## Abbreviations

5-ASA: 5-Aminosalicylic acid
ASV: Amplicon sequencing variant
DAI: Disease activity index
DSS: Dextran sulfate sodium
FMT: Fecal microbiota transfer
GF: Germ-free
H&E: Hematoxylin and eosin
HPLC: High-performance liquid chromatography
IBD: Inflammatory bowel disease
IL: Interleukin
MLN: Mesenteric lymph nodes
*N*-acetyl-5-ASA: 5-Acetylamino-2-hydroxybenzoic acid
NF-κB: Nuclear factor kappa-light-chain-enhancer of activated B cells
NT: Non-treated
PBS: Phosphate-buffered saline
PCoA: Principal coordinate analysis
SPF: Specific-pathogen-free
TEM: Transmission electron microscopy
TGF: Transforming growth factor
TNF-α: Tumor necrosis factor-alpha
Treg: Regulatory T cells
UC: Ulcerative colitis
