# Supplementary material for: 5-Aminosalicylic acid alters the gut microbiota and altered microbiota transmitted vertically to offspring have protective effects against colitis
Source: Sci Rep. 2023 Jul 28;13:12241. doi: 10.1038/s41598-023-39491-x (PMC10382598; doi:10.1038/s41598-023-39491-x)
Supplement: Supplementary file 1 — Supplementary Information. [file 41598_2023_39491_MOESM1_ESM.docx]

**Supplemental materials (information)**

**5-aminosalicylic acid alters the gut microbiota and altered microbiota transmitted vertically to offspring have protective effects against colitis**

**Haruka Wada, Jun Miyoshi, Satoshi Kuronuma, Yuu Nishinarita, Noriaki Oguri, Noritaka Hibi, Osamu Takeuchi, Yoshihiro Akimoto, Sonny T.M. Lee, Minoru Matsuura, Taku Kobayashi, Toshifumi Hibi, Tadakazu Hisamatsu**


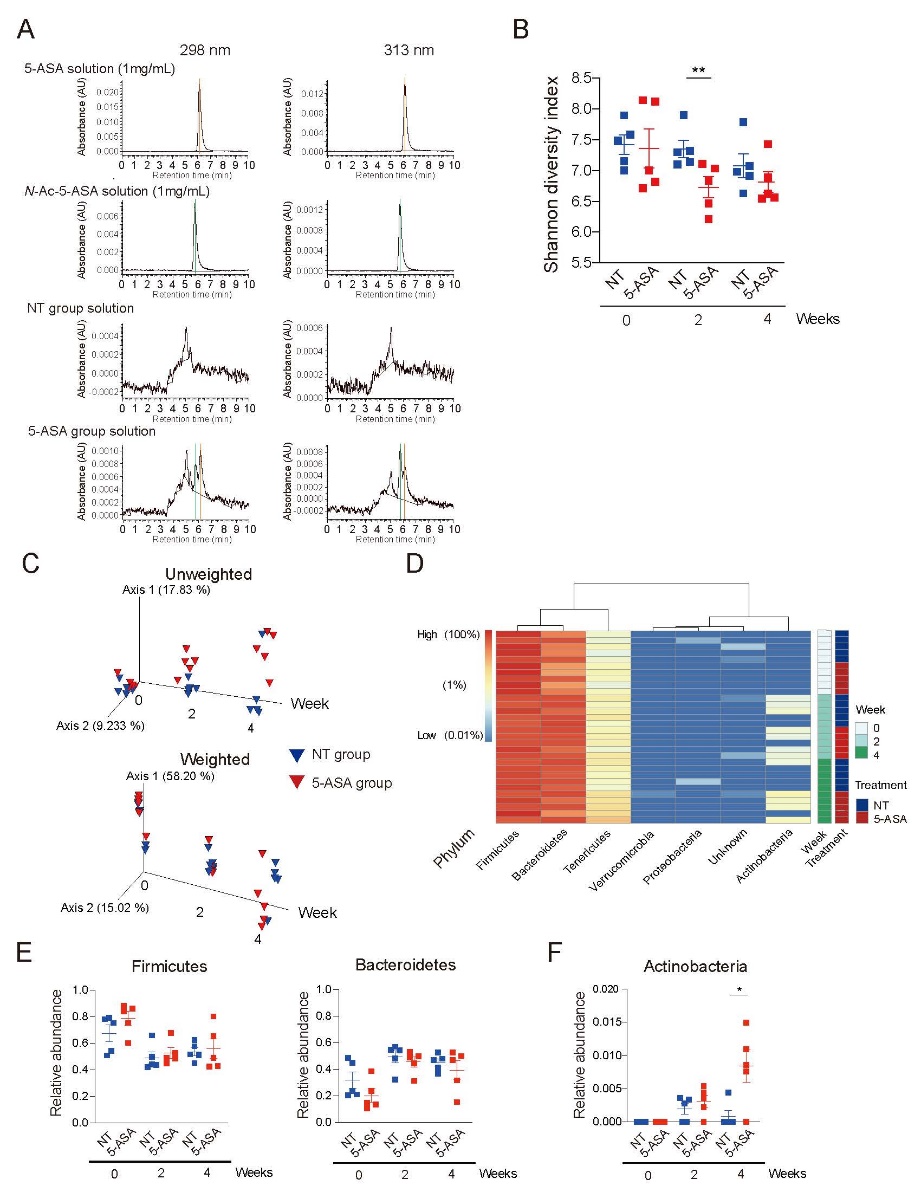
Supplemental Figure S1. Oral administration of 5-aminosalicylic acid alters the gut bacterial composition regardless of sex.

(A) High-performance liquid chromatography (HPLC) was performed using cecal contents to determine the presence of 5-aminosalicylic acid (5-ASA) and *N*-acetyl-5-ASA. Peaks compatible with 5-ASA and *N*-acetyl-5-ASA were detected in the 5-ASA group but not in the non-treated (NT) group. (B) Changes in the Shannon diversity index over time in 5-ASA-treated male animals. (C) PCoA plots based on unweighted and weighted UniFrac distances of the intestinal bacterial compositions in male animals. (D) Heatmap of the relative abundances of bacterial phyla over time in male animals. Each row represents a single DNA sample, and each column represents each phylum. (E) Changes in the relative abundances of phyla Firmicutes and Bacteroidetes over time in male animals. (F) Changes in the relative abundance of phylum Actinobacteria over time in male animals. **p* < 0.05, ***p* < 0.01, Mann-Whitney *U*-test. The data are the mean ± SEM.


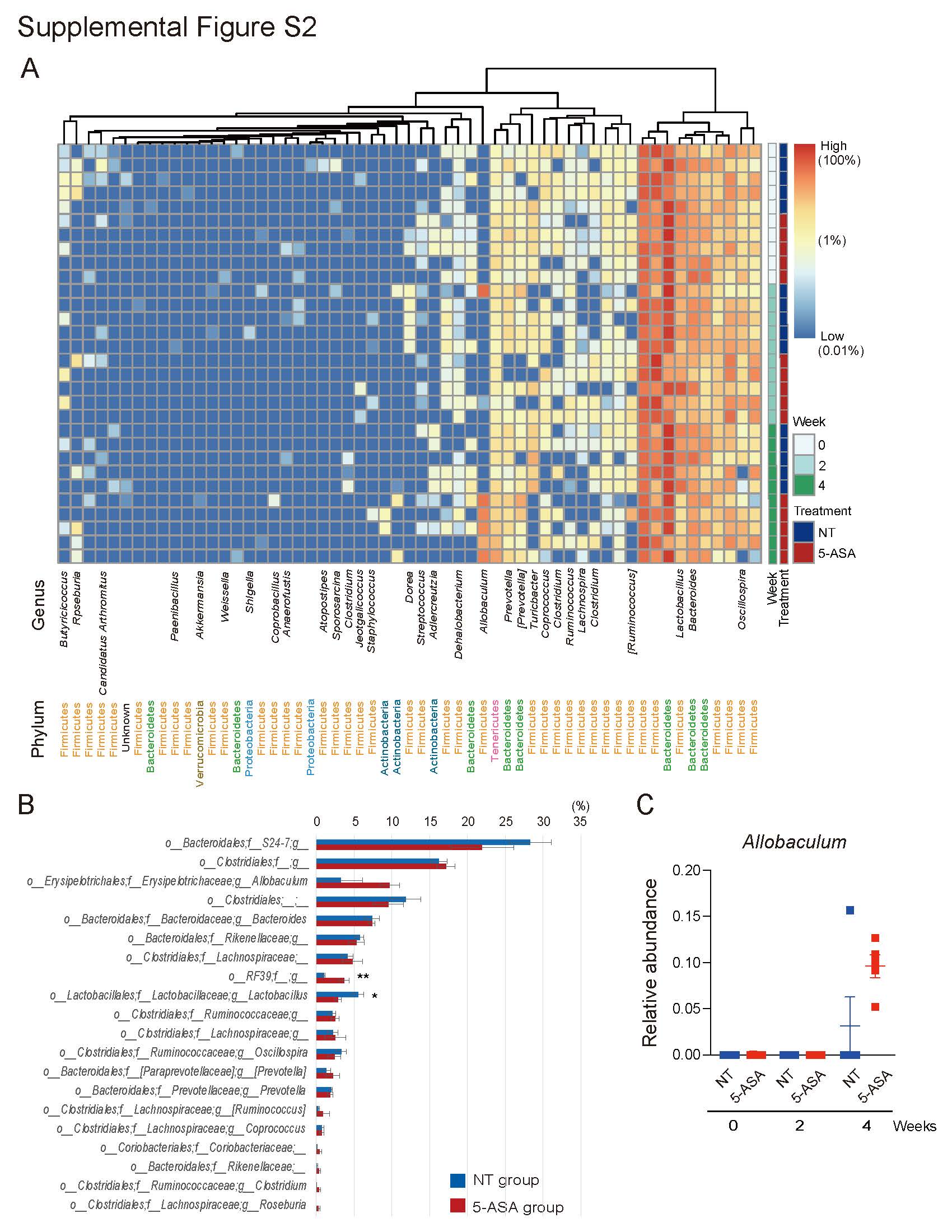


Supplemental Figure S2. Bacterial genera in the intestinal microbiota are altered by 5-aminosalicylic acid administration regardless of sex.

(A) Heatmap of the relative abundances of bacterial genera over time in male animals. Each row represents a single DNA sample, and each column represents each genus. A blank in the name of the genus indicates it was not annotated with QIIME 2. (B) Top 20 bacterial genera with high relative abundance in male animals treated with 5-aminosalicylic acid (5-ASA). (C) Changes in the relative abundance of genus *Allobaculum* in male animals. **p* < 0.05, ***p* < 0.01, Mann-Whitney *U*-test. The data are the mean ± SEM.


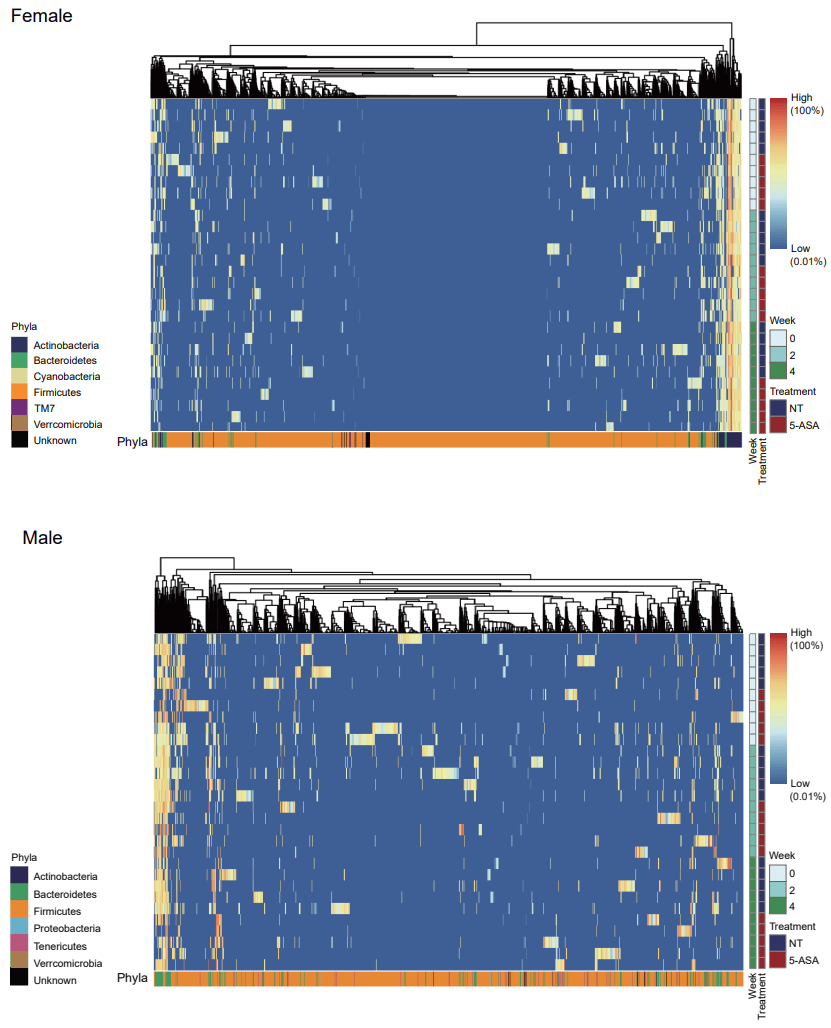


Supplemental Figure S3. Heatmaps of the relative abundances of 16S rRNA gene amplicon sequencing variants over time in female and male animals.

Each row represents a single DNA sample and each column represents each ASV.


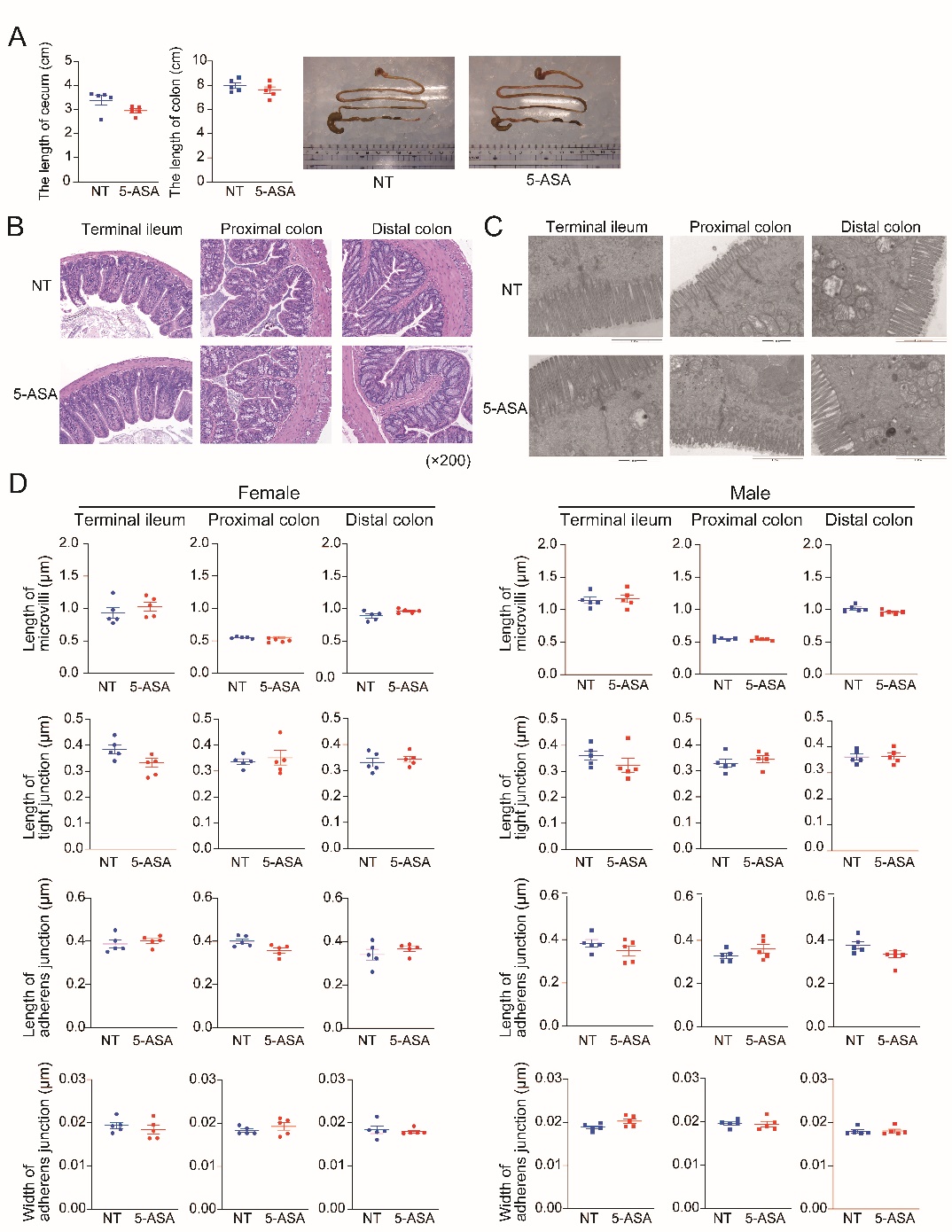
Supplemental Figure S4. Oral 5-aminosalicylic acid administration does not affect the intestinal morphology regardless of sex.

(A) The length of the cecum and colon in male animals in the 5-aminosalicylic acid (5-ASA) group and non-treated (NT) group. Representative images are presented. (B) Representative images of male intestinal specimens stained with hematoxylin and eosin (H&E) under an optical microscope (×200). (C) Representative images of male intestinal specimens obtained by transmission electron microscopy (D). Scale bars, 1 µm. There were no significant differences in the length of microvilli, length of tight junctions, and length and width of adherens junctions between either sex in the 5-ASA and NT groups. The data are the mean ± SEM.


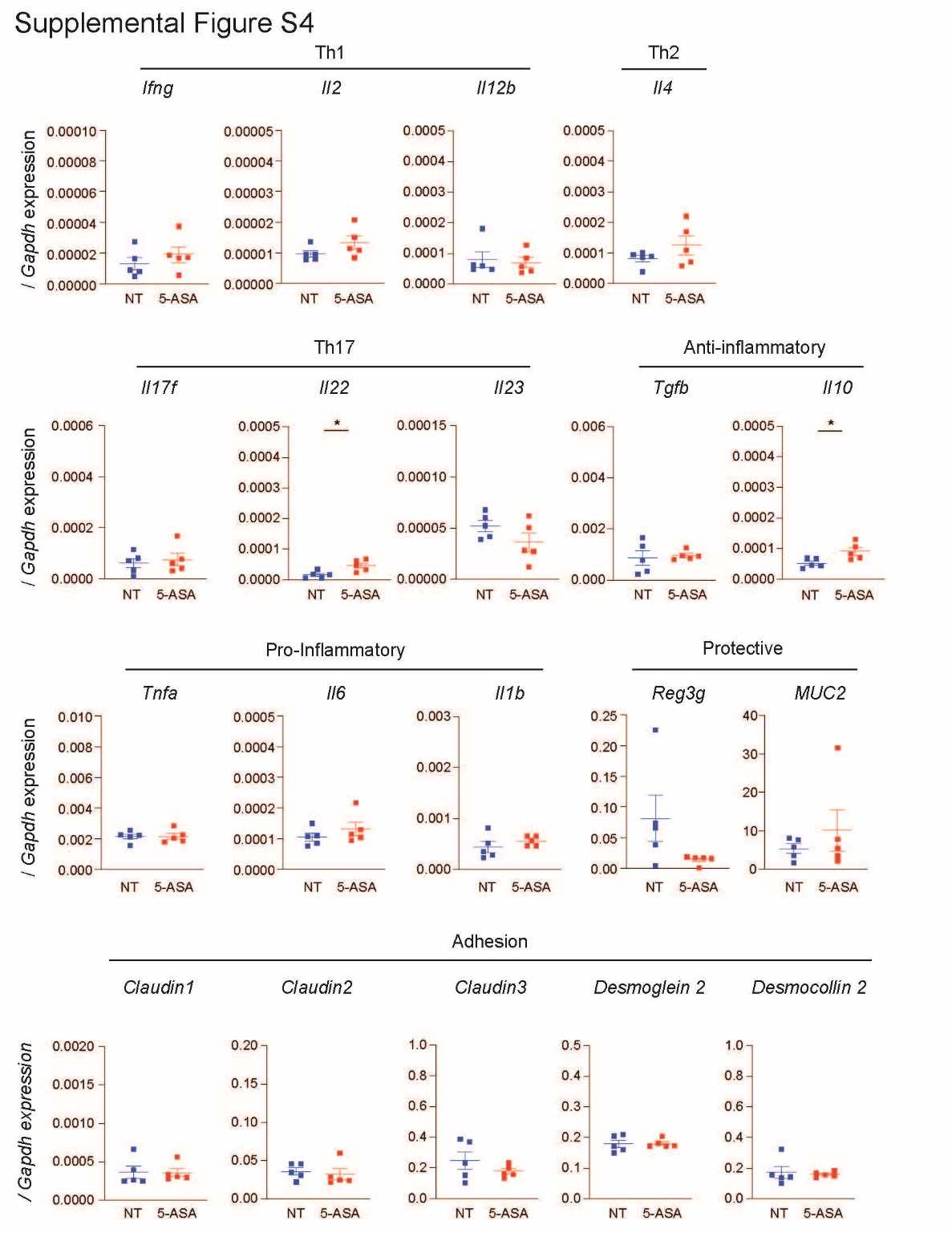
Supplemental Figure S5. Oral administration of 5-aminosalicylic acid influences the mRNA expression in the colonic mucosa regardless of sex.

mRNA expressions of cytokines and pro- and anti-inflammatory molecules involved in colonic inflammation in the colonic mucosa were examined in the 5-aminosalicylic acid (5-ASA) group and non-treated (NT) group by real-time qPCR. mRNA expressions are expressed as ΔΔCT relative to the housekeeper gene *Gapdh*. Male data are presented as the mean ± SEM. **p* < 0.05, Mann-Whitney *U*-test.


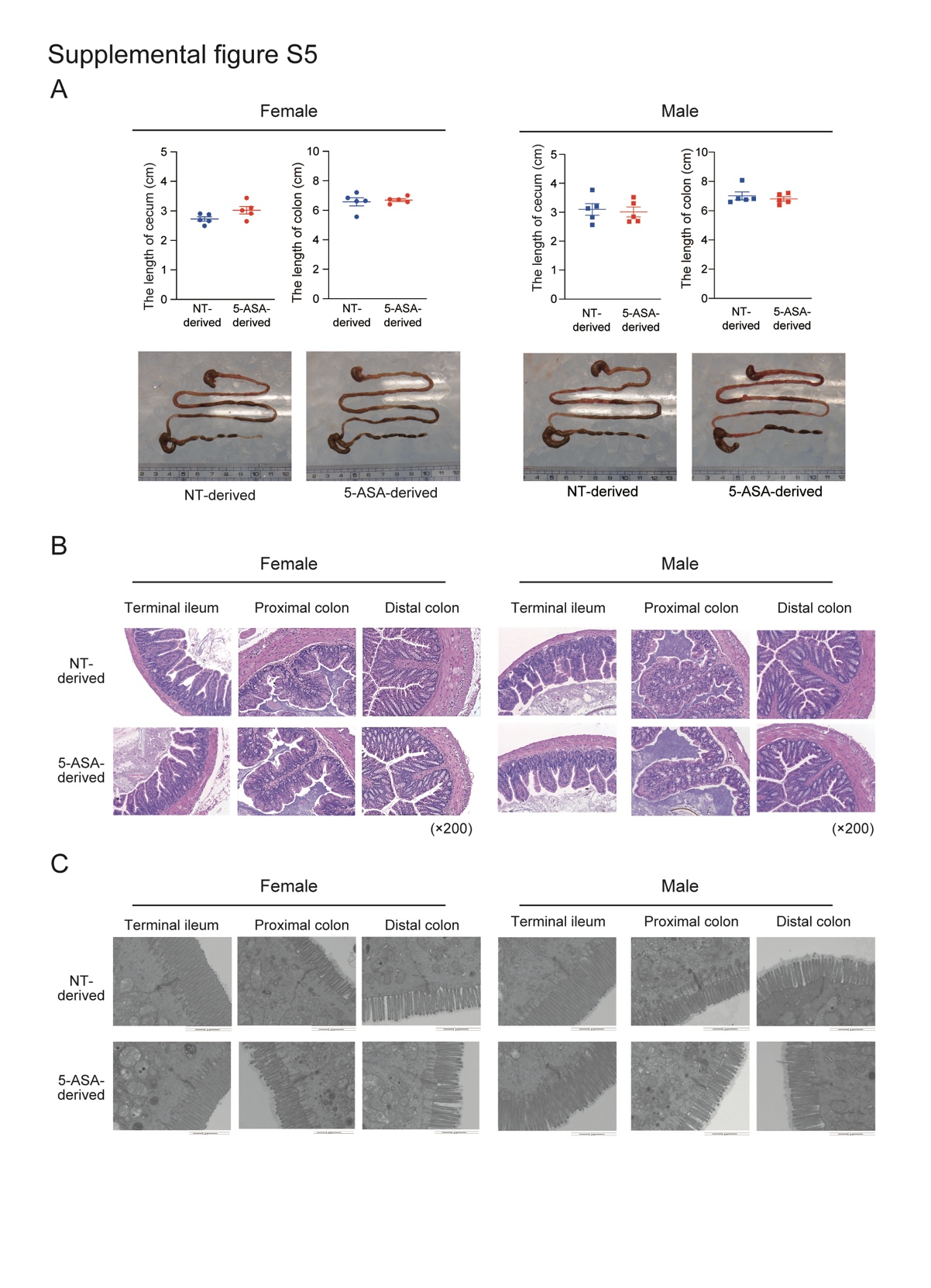
Supplemental Figure S6. Alterations in the intestinal microbiota by 5-aminosalicylic acid vertically transmitted to offspring do not affect intestinal morphology regardless of sex.

(A) The length of the cecum and colon in animals in the 5-aminosalicylic acid (5-ASA)-derived group and non-treated (NT)-derived group (n=5 of each sex). Representative images are presented. (B) Representative images of intestinal specimens stained with hematoxylin and eosin (H&E) under an optical microscope (×200). (C) Representative images of intestinal specimens obtained by transmission electron microscopy. Scale bars, 1 µm. The data are the mean ± SEM.


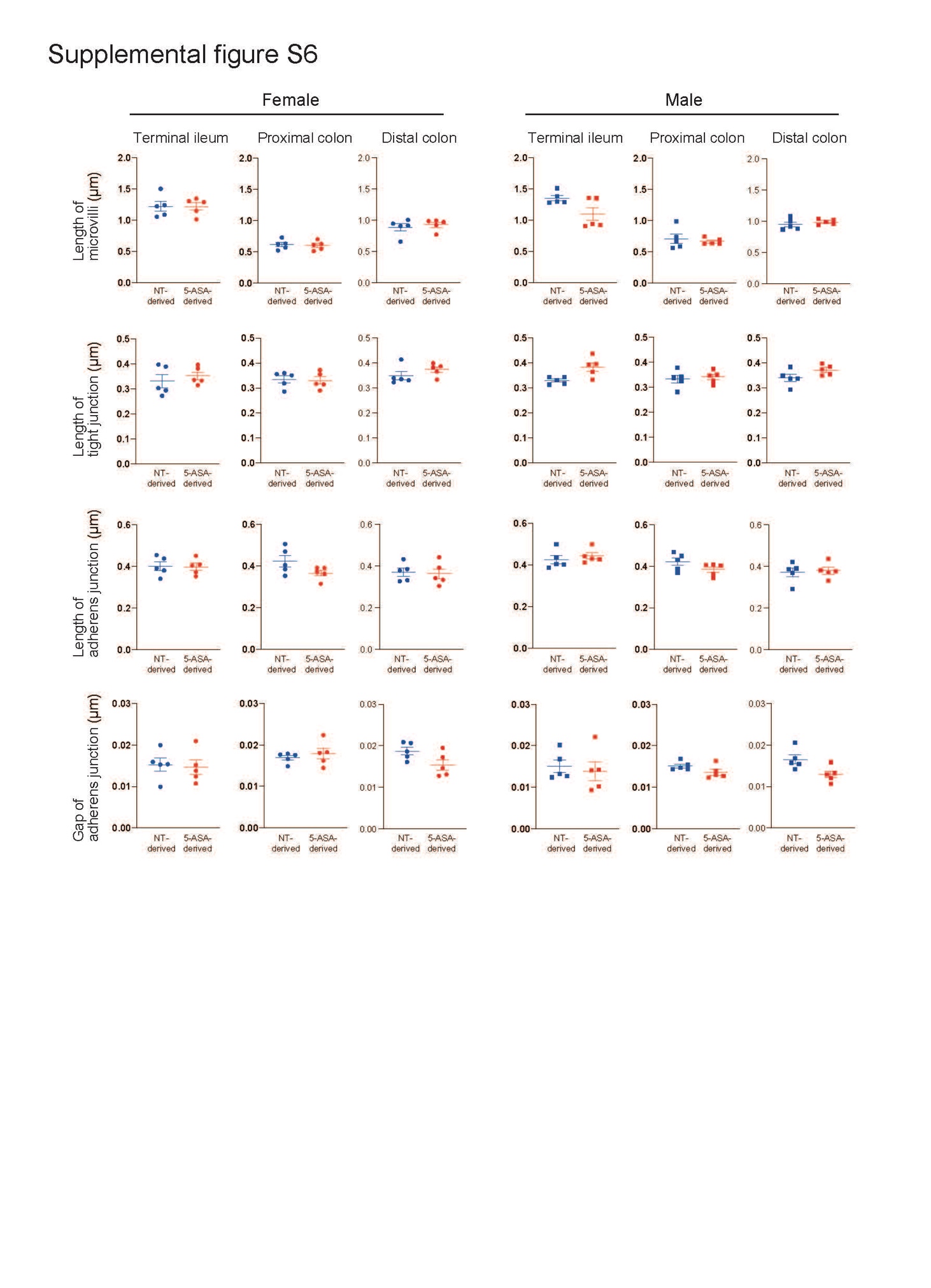


Supplemental Figure S7. Alterations in the intestinal microbiota by 5-aminosalicylic acid vertically transmitted to offspring do not affect the ultrastructure of the terminal ileum and colon.

There were no significant differences in the length of microvilli, length of tight junctions, and length and width of adherens junctions between either sex in the 5-ASA-derived and NT-derived groups (n=5 of each sex). The data are the mean ± SEM.


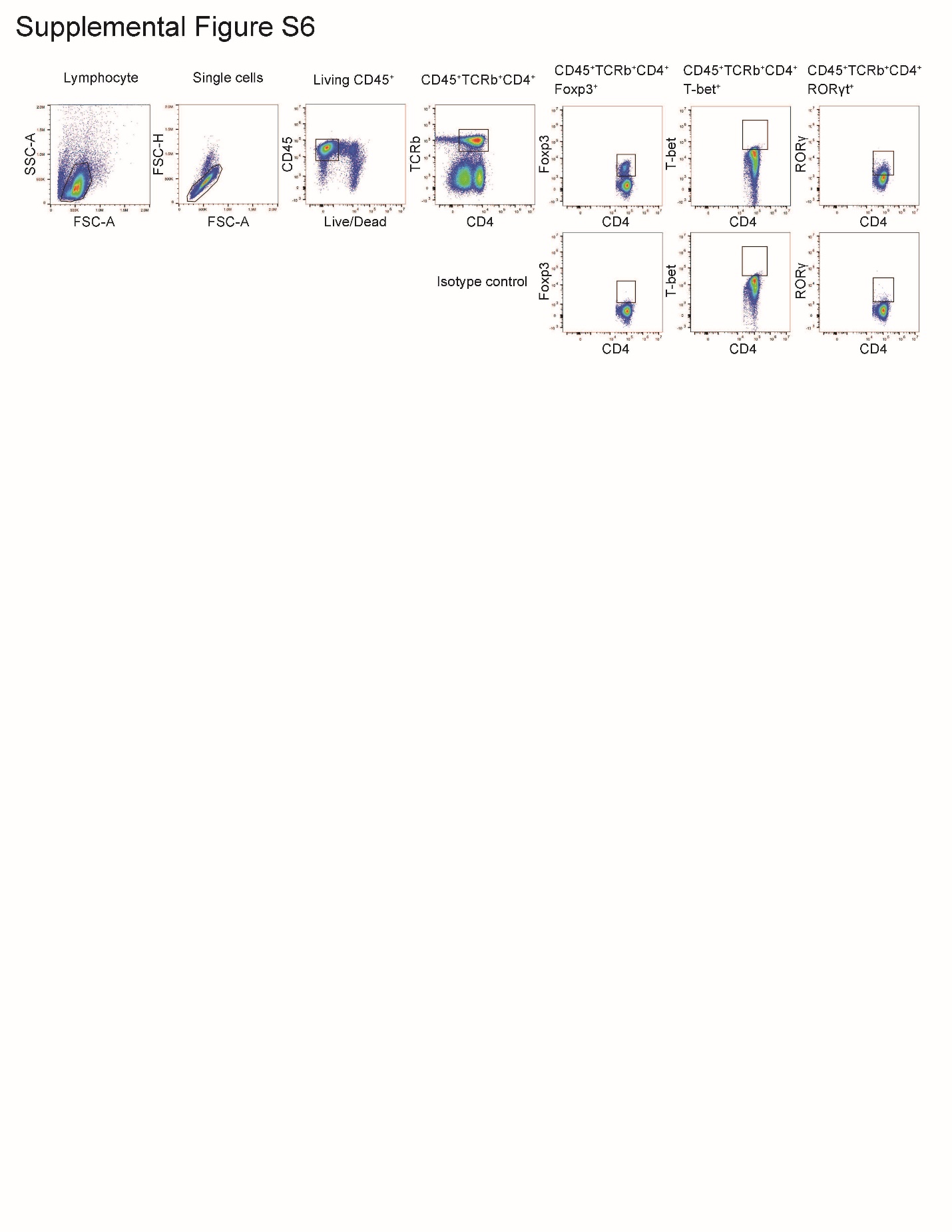


Supplemental Figure S8. Flow cytometry gating strategy.

Representative images of the flow cytometry gating strategy for analyzing T cell populations in the mesenteric lymph nodes with representative isotype controls.


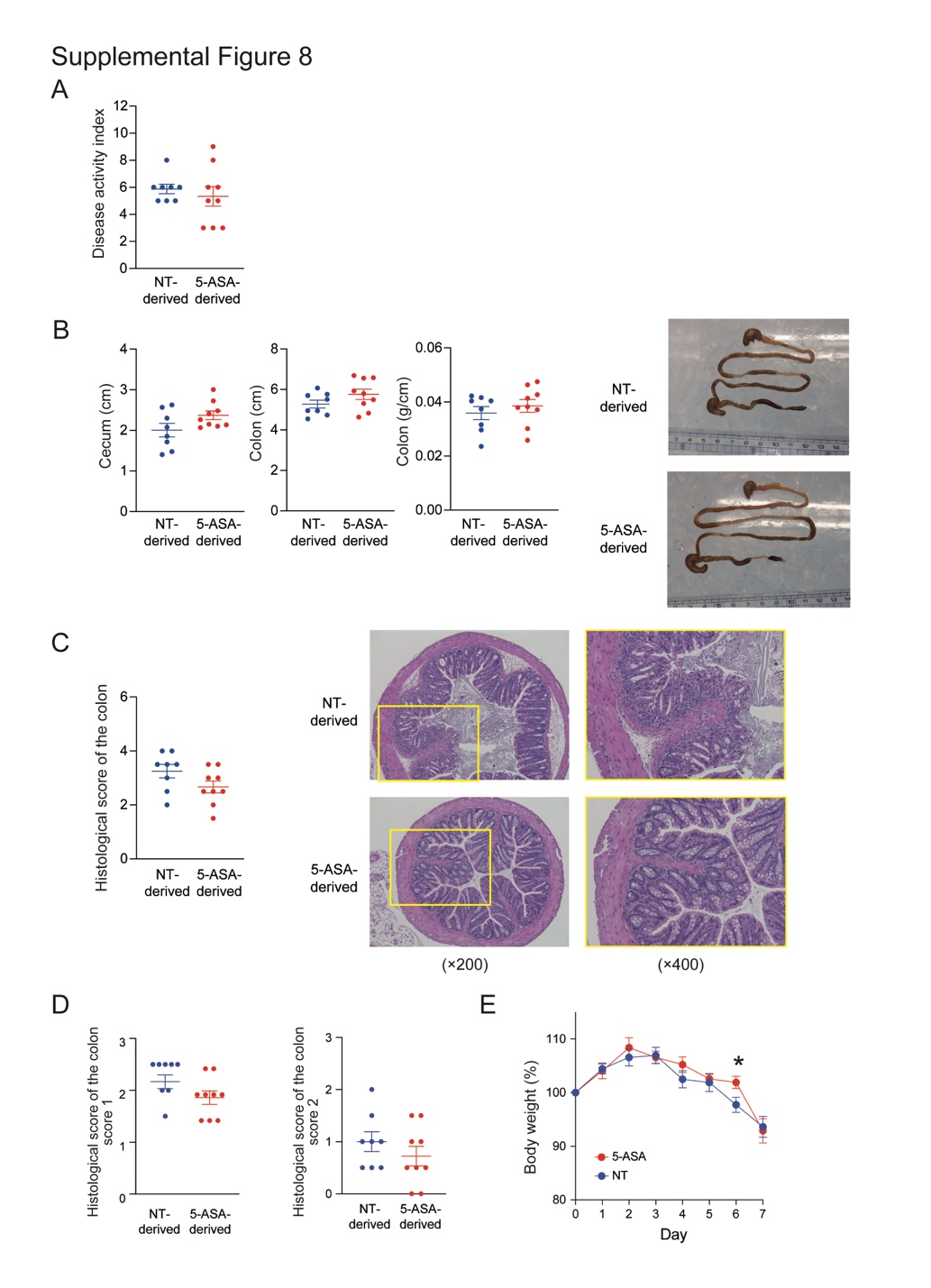
Supplemental Figure S9. Vertically transmitted intestinal microbiota altered by 5-aminosalicylic acid tended to be protective against experimental colitis in female offspring, but the protective effect was lower compared with that in male animals.

The severity of colitis was assessed on day 7 after dextran sulfate sodium (DSS)-induced colitis was initiated in the 5-aminosalicylic acid (5-ASA)-derived group and non-treated (NT)-derived group. (A) Disease activity index. (B) The length of the cecum, length of the colon, and the colon weight per length with representative macroscopic images. (C) Histological score of the colon with representative microscopic images (×200 and ×400). (D) Histological scores of score 1 (inflammatory cell infiltrate) and score 2 (intestinal architecture). (E) Body weight changes during DSS treatment. The data are the mean ± SEM.

Supplemental Table

Supplemental Table 1. Histological scoring of colitis

| Inflammatory cell infiltrate | | Score 1 |
| --- | --- | --- |
| Severity | Extent |  |
| Mild | Mucosa | 1 |
| Moderate | Mucosa and submucosa | 2 |
| Marked | Transmural | 3 |
| Intestinal architecture | | Score 2 |
| Epithelial changes | Mucosal architecture |  |
| Focal erosions |  | 1 |
| Erosions | ±Focal ulcerations | 2 |
|  | Extended ulcerations ± granulation tissue ± pseudopolyps | 3 |
|  | Sum of scores 1 and 2 | 0 - 6 |

Supplemental Table 2. Disease activity index (DAI) score

| Score | Weight loss | Stool consistency | Bleeding |
| --- | --- | --- | --- |
| 0 | None | Normal | No bleeding |
| 1 | 1%–5 % | – | – |
| 2 | 5%–10 % | Loose stools | Slight bleeding |
| 3 | 10%–15 % | – | – |
| 4 | Greater than 15% | Watery diarrhea | Gross bleeding |

Supplemental Table 3. Primers for reverse-transcription quantitative PCR

| Gene |  |  | Sequence |  |
| --- | --- | --- | --- | --- |
| *Ifng* | Forward | 5' | ATGAACGCTACACACTGCATC | 3' |
|  | Reverse | 5' | CCATCCTTTTGCCAGTTCCTC | 3' |
| *Il2* | Forward | 5' | TGAGCAGGATGGAGAATTACAGG | 3' |
|  | Reverse | 5' | GTCCAAGTTCATCTTCTAGGCAC | 3' |
| *Il12b* | Forward | 5' | TGGTTTGCCATCGTTTTGCTG | 3' |
|  | Reverse | 5' | ACAGGTGAGGTTCACTGTTTCT | 3' |
| *Il4* | Forward | 5' | GGTCTCAACCCCCAGCTAGT | 3' |
|  | Reverse | 5' | GCCGATGATCTCTCTCAAGTGAT | 3' |
| *Il17f* | Forward | 5' | TGCTACTGTTGATGTTGGGAC | 3' |
|  | Reverse | 5' | AATGCCCTGGTTTTGGTTGAA | 3' |
| *Il22* | Forward | 5' | ATGAGTTTTTCCCTTATGGGGAC | 3' |
|  | Reverse | 5' | GCTGGAAGTTGGACACCTCAA | 3' |
| *Il23a* | Forward | 5' | ATGCTGGATTGCAGAGCAGTA | 3' |
|  | Reverse | 5' | ACGGGGCACATTATTTTTAGTCT | 3' |
| *Tgfb* | Forward | 5' | CTCCCGTGGCTTCTAGTGC | 3' |
|  | Reverse | 5' | GCCTTAGTTTGGACAGGATCTG | 3' |
| *Il10* | Forward | 5' | GCTCTTACTGACTGGCATGAG | 3' |
|  | Reverse | 5' | CGCAGCTCTAGGAGCATGTG | 3' |
| *Tnfa* | Forward | 5' | CCCTCACACTCAGATCATCTTCT | 3' |
|  | Reverse | 5' | GCTACGACGTGGGCTACAG | 3' |
| *Il6* | Forward | 5' | TAGTCCTTCCTACCCCAATTTCC | 3' |
|  | Reverse | 5' | TTGGTCCTTAGCCACTCCTTC | 3' |
| *Il1b* | Forward | 5' | GCAACTGTTCCTGAACTCAACT | 3' |
|  | Reverse | 5' | ATCTTTTGGGGTCCGTCAACT | 3' |
| *Reg3g* | Forward | 5' | ATGCTTCCCCGTATAACCATCA | 3' |
|  | Reverse | 5' | GGCCATATCTGCATCATACCAG | 3' |
| *Muc2* | Forward | 5' | ATGCCCACCTCCTCAAAGAC | 3' |
|  | Reverse | 5' | GTAGTTTCCGTTGGAACAGTGAA | 3' |
| *Claudin1* | Forward | 5' | GGGGACAACATCGTGACCG | 3' |
|  | Reverse | 5' | AGGAGTCGAAGACTTTGCACT | 3' |
| *Claudin2* | Forward | 5' | CAACTGGTGGGCTACATCCTA | 3' |
|  | Reverse | 5' | CCCTTGGAAAAGCCAACCG | 3' |
| *Claudin3* | Forward | 5' | ACCAACTGCGTACAAGACGAG | 3' |
|  | Reverse | 5' | CAGAGCCGCCAACAGGAAA | 3' |
| *Desmoglein2* | Forward | 5' | GTGGTCTGCTTGGACTTTGGA | 3' |
|  | Reverse | 5' | GGAACGGTTTGCCTTCATTTC | 3' |
| *Desmocollin2* | Forward | 5' | ATGGCGGCTGTGGGATCTAT | 3' |
|  | Reverse | 5' | GCAAGGATCGCAAGGGTCAA | 3' |
| *Gapdh* | Forward | 5' | AGGTCGGTGTGAACGGATTTG | 3' |
|  | Reverse | 5' | TGTAGACCATGTAGTTGAGGTCA | 3' |
